# Correlation between the vascular resistance index and arteriography for assessment of the distal arterial bed in chronic limb threatening ischemia

**DOI:** 10.1590/1677-5449.202300712

**Published:** 2024-02-05

**Authors:** Gabriela de Oliveira Buril, Esdras Marques Lins, Emmanuelle Tenório Albuquerque Godoi Berenguer de Barros e Silva, Fernanda Appolônio da Rocha, Juliana Cavalcanti de Siqueira Charamba, Rebecca Paes de Andrade Souza Caldas, Isadora Ísis Fernandes Vieira, Paloma Karine Araújo da Silva

**Affiliations:** 1 Universidade Federal de Pernambuco - UFPE, Hospital das Clínicas - HC, Recife, PE, Brasil.; 2 Universidade Federal de Pernambuco - UFPE, Centro de Ciências Médicas - CCM, Recife, PE, Brasil.

**Keywords:** chronic limb-threatening ischemia, vascular resistance, Doppler ultrasound, peripheral arterial disease, lower limbs

## Abstract

**Background:**

Patients with chronic limb threatening ischemia (CLTI) of the lower limbs (LL) undergo arteriography for revascularization surgery planning. Doppler ultrasound (DU) is non-invasive and can provide information about the distal arteries through measurement of the resistance index (RI).

**Objectives:**

To correlate the Rutherford Angiographic Classification with the RI for assessment of the distal arterial bed of the LL.

**Methods:**

A cross-sectional study, conducted at a public tertiary hospital with 120 patients with LL CLTI, from September 2019 to April 2022. The RI of arteries that were candidates for revascularization was compared with the images of the same arteries obtained using arteriography, using the Rutherford Angiographic Classification of the distal bed.

**Results:**

A total of 120 LL were assessed in 120 patients with a mean age of 68.6 years. The sample was 50.0% male and 90.0% of the patients in the sample were classified as Rutherford category five. The RI values found for the arteries of the leg exhibited a statistically significant positive correlation with the Rutherford Classification (anterior tibial, p< 0.01; posterior tibial, p = 0.012 fibular, p = 0.034; and dorsalis pedis, p < 0.001).

**Conclusions:**

In this study, RIs for the arteries of the leg measured using Doppler ultrasound exhibited a positive correlation with the Rutherford Classification. This index could be useful for assessment of the distal arterial bed of the lower limbs of patients with chronic limb threatening ischemia.

## INTRODUCTION

Many different tests are available for assessing the distal arteries of the lower limbs (LL) in peripheral arterial occlusive disease (PAOD). Ultrasonography with Doppler (DU) is generally the first examination used to assess patients with PAOD.^1^ Subsequently, an examination using a contrast medium may be needed to conduct a more detailed evaluation of lesion anatomy, specifically: angiotomography, magnetic resonance angiography, or digital subtraction angiography (DSA).^2^

Currently, DSA remains the gold standard examination for planning LL revascularization surgery. However, DSA is an invasive examination, uses iodinated contrast, exposes the patient and medical team to ionizing radiation, and is expensive, in addition to having limitations, such as visualization of the arterial bed distal of an occlusion which can be inadequate.^2-4^ Visualization of the distal bed may be difficult if blood flow is severely restricted, whether because of multiple and extensive occlusions, insufficient cardiac output, or intolerable ischemic pain that prevents the patient from remaining immobile during injection of the contrast. In such situations, visualization of the arterial bed of the leg and foot can be considerably compromised.^5,6^

Using DSA findings, Rutherford proposed a classification for assessment of the distal arterial bed (DAB) for planning revascularization surgery. It scores each distal artery for anastomosis site (the point to which the artery is patent) multiplied by the appearance of the same artery downstream, summing this to a score for runoff to the foot via the plantar arch (PA). A good distal bed is one in which the artery is patent from the site under assessment to a complete PA. For example, considering an anterior tibial artery (AT) used for anastomosis at its distal third (value = 2), patent to where it runs off at the foot, with no stenosis exceeding 20% (value = 0), a pedal arterial bed with continuous communication to a patent vessel (value = 1), and an incomplete PA (value = 2), the score calculated for the AT would be as follows: **(2 x 0) + (1 x 2) + 1 = 3**.^7^

Doppler ultrasound has become very useful for evaluation of PAOD of the LL, yielding more and more information. In addition to being low-cost, noninvasive, free from exposure to radiation, and not needing nephrotoxic contrast, DU can also be used to assess the artery wall and classify it in terms of thickness and calcium content. In addition to local assessment, DU can also be used to assess the distal bed, by measuring the resistance index (RI), which estimates peripheral vascular resistance status, i.e., it assesses the resistance to flow through the artery being studied. The lower the RI, the more open the distal trunk arteries (and the better the DAB) and the lower the resistance to flow after revascularization. Doppler ultrasound also offers the advantage of indicating the resistance of a given artery, without necessarily having morphologically assessed the entire distal bed. The main disadvantage of DU is the difficulty of conducting anatomical assessment of all of the vessels that comprise the plantar arch arteries.^1^ The RI is calculated as the ratio of the difference between peak systolic velocity and end-diastolic velocity, divided by peak systolic velocity, and indirectly assesses what can be seen on DSA.^1,4^

There is consensus in the global literature that low peripheral vascular resistance is one of the factors that maintain an arterial bypass or angioplasty open. However, there are few studies using DU to assess vascular resistance.^5^ Considering that PAOD of the LL is a growing public health problem, imposing a significant cost burden on patients and health care systems, there is a need to study less invasive and lower cost examinations that can facilitate decision-making when choosing the best treatment for patients, including by suggesting which arteries should be revascularized.^6-18^

The objective of this study was to correlate the Rutherford Angiographic Classification with RI of the distal arteries of the LL.

## METHOD

The study enrolled 120 patients admitted to the vascular surgery wards at the Hospital das Clínicas/Empresa Brasileira de Serviço Hospitalares da Universidade Federal de Pernambuco (HC/EBSERH-UFPE) with CLTI of the LL (Rutherford grades 4, 5, and 6^7^ ) who underwent DU and DSA of the arteries of diseased limbs for preoperative assessment preparatory to revascularization surgery from August 2019 to April 2022. The study design was cross-sectional, with prospective data collection, and sampling was by convenience, assessing all patients admitted with CLTI of the LL during the preoperative period of revascularization surgery.

Sample selection was by census, enrolling all patients admitted during the study period with PAOD and CLTI of the LL.

All patients were analyzed using the Rutherford PAOD Classification.^7^ Next, patients were examined with DU and then with DSA of the LL arteries. The assessments with DU and analysis of the angiographic images were all performed by a single examiner.

During DU, each leg artery that was a candidate recipient for arterial bypass or angioplasty was assessed for patency/occlusion, stenosis, calcifications, and wave velocity morphology, in addition to measurement of peak systolic velocity (VPS), end-diastolic velocity (VDF), and RI. The site at which RI was measured corresponded to the point at which the lumen of the distal recipient artery was best, or as distal as possible. The RI values for the arterial bed distal of an occlusion can vary from 0 to 1.0, where 0 corresponds to a continuous flow wave and 1.0 corresponds to a high-resistance wave, with no diastolic flow (the worst DAB).^17,18^

Arteriography images were assessed by a single observer. The angiographic images of the distal bed were classified as per Rutherford, to convert a qualitative analysis (image) into a quantitative variable. Each artery was assessed individually, but not all patients had all three arteries of the leg patent, so the n for each artery was different and less than 120 (Tables 1 and 2).^7^

**Table 1:** Weighting of arterial runoff (total of three units) - local.^7^

**Site of distal anastomosis**	**Number of units attributed**		
**(artery)**	**3**	**2**	**1**
Common iliac		External iliac	Internal iliac
External iliac	Common femoral	Femoral	Deep
Common femoral		Femoral	Deep
Popliteal above the knee	Distal popliteal		Anterior tibial
Popliteal below the knee			Posterior tibial
			Fibular
Anterior tibial		Distal tibial	Plantar arch
Posterior tibial		Distal tibial	Plantar arch
Fibular		Pedal runoff	Collaterals to the tibial
Dorsalis pedis/inframalleolar			arteries

**Table 2:** Weighting of arterial runoff (total of three points) - occlusion.^7^

**Degree of occlusion**	**Number of points scored per unit**				
	**3**	**2.5**	**2**	**1**	**0**
Major runoff vessels	Occluded along entire length	Occluded for less than ½ of length; visible collaterals	Maior stenosis from 50% to 99%	Maior stenosis from 20% to 49%	Major stenosis less than 20%
Pedal runoff	No patent pedal arteries	Partially pervious or totally pervious after a subocclusive lesion	Communication with patent vessel, but incomplete arch	One or more subocclusive stenoses	Pedal runoff totally patent (stenosis <20%)

The value attributed to each artery was the weighting for the potential anastomosis site (where the artery was patent) multiplied by the appearance of the same artery downstream. This is added to the weighting for pedal runoff - at the plantar arch (PA). For example, considering an anterior tibial artery (AT) used for anastomosis at its distal third (value = 2), patent to where it runs off at the foot, with no stenosis exceeding 20% (value = 0), and pedal runoff with continuous communication to a patent vessel, the dorsalis pedis artery (value = 1), but an incomplete PA (value = 2), the score calculated would be as follows: AT = (2 x 0) + (1 x 2) + 1 = 3. All equations end with addition of one point, so that results are always greater than zero. Scores for runoff for the anterior tibial, posterior tibial, and fibular arteries varied from 10 to 1, where higher values indicate worse runoff. Scores for the dorsalis pedis artery vary from 7 to 1.^7^

The runoff of the AT, posterior tibial (PT), and fibular (FIB) arteries were scored from 10 to 1, where higher scores indicate worse runoff. Runoff scores for the dorsalis pedis (DP) artery vary from 7 to 1.^7^ The present study did not include an assessment of the best artery for revascularization in isolation. All of the distal arteries were studied and their respective RI measured.

Results were analyzed using SPSS 13.0 (Statistical Package for the Social Sciences) for Windows and Excel 2010. All tests were calculated for a 95% confidence interval. Results were analyzed on the basis of valid responses, i.e. unanswered questions were not included in the calculations. Spearman’s rho test was used to correlate RI (0.0 to 1.0)^17^ with variables from the Rutherford Classification (runoff scores for the AT, PT, and FIB arteries ranged from 10 to 1, where higher scores mean worse runoff, and scores for the DP artery ranged from 7 to 1).^7^ Numerical variables were expressed as measures of central tendency and dispersion. The Kolmogorov-Smirnov test of normality was used for quantitative variables.

The study was approved by the Research Ethics Committee at the HC/EBSERH-UFPE, under registration number 3.471.560.

## RESULTS

A total of 120 LL were evaluated in 120 patients aged from 32 to 96 years (mean age of 68.6 years, with standard deviation of 10.3 years), all with PAOD secondary to atherosclerosis. The sample included 60 (50.0%) male patients. A total of 108 (90.0%) patients were classified as Rutherford category 5 (Table 3).

**Table 3:** Characteristics of the sample of 120 patients.

**Characteristics**	**Value**
**Demogr**ap**hic factors**	
Male sex	60 (50.0%)
Age	68.6 ± 10.3
**Risk factors**	
SAH	108 (90.0%)
DM	92 (76.7%)
Smoking	61 (50.8%)
CKF HD	8 (6.7%)
Rutherford Classification **(**PAOD**)**	
4	3 (2.5%)
5	109 (90.8%)
6	8 (6.7%)
**Prior amputation**	21 (17.5%)
**Type of lesion**	
Spontaneous	97 (80.8%)
Traumatic	19 (15.8%)

SAH = systemic arterial hypertension; DM = diabetes mellitus*;* CKF = chronic kidney failure; HD = hemodialysis; PAOD = peripheral atherosclerotic occlusive disease.

Of the comorbidities analyzed, systemic arterial hypertension (SAH) was present in 108 patients (90.0%); DM in 92 patients (76.7%); and smoking in 61 patients (50.8%), while 6.7% of the patients had end-stage chronic kidney failure requiring hemodialysis.

With regard to amputations, 21 (17.5%) patients had had some type of prior amputation; while injuries to the extremities were classified as spontaneous in 100 patients (83.4%).

Table 3 shows the characteristics of the study sample. Table 4 shows the distribution of Rutherford Classification^7^ distal bed scores for the 120 patients.

**Table 4:** Classification of the arteries of the distal bed, according to the Rutherford Classification.

**Arteries (n)**	**Mean ± Standard deviation**	**Median (Q1; Q3)**	**Minimum - Maximum**
AT (42)	3.57 ± 1.92	3.00 (3.00; 4.25)	1.00 - 9.00
PT (44)	3.05 ± 1.61	3.00 (2.25; 3.00)	1.00 - 7.50
FIB (64)	2.93 ± 1.36	3.00 (3.00; 3.00)	1.00 - 6.50
DP (48)	2.44 ± 1.05	3.00 (1.00; 3.00)	1.00 - 5.00

AT = anterior tibial; PT = posterior tibial; FIB = fibular; DP = dorsalis pedis.

The RI of distal arteries was measured for all patent arteries with possibilities for revascularization. Table 5 shows the mean, median, and range for the RI observed for the AT, PT, FIB, and DP arteries.

**Table 5:** Resistance indices measured for the anterior tibial, posterior tibial, fibular, and dorsalis pedis.

RI **(n)**	**Mean ± SD**	**Median (Q1; Q3)**	**Minimum - maximum**
AT (42)	0.60 ± 0.23	0.57 (0.45; 0.76)	0.20 - 1.00
PT (44)	0.58 ± 0.18	0.56 (0.47; 0.72)	0.19 - 0.88
FIB (64)	0.62 ± 0.20	0.65 (0.49; 0.75)	0.16 - 1.00
SD (48)	0.52 ± 0.19	0.51 (0.39; 0.63)	0.15 - 1.00

RI = resistance index; AT = anterior tibial; PT = posterior tibial; FIB = fibular; PD = dorsalis pedis; SD = standard deviation.

Next, the angiographic scores for each artery were correlated with their respective RI. Table 6 shows the correlation between the Rutherford classification for the AT, PT, FIB, and DP arteries and their respective RI. The Spearman rho correlation test was used to analyze correlations between variables.

**Table 6:** Coefficients for correlations between the Rutherford Classification and resistance indices for the tibial, fibular, and dorsalis pedis arteries.

	**Correlation coefficient ^A^**
Anterior tibial artery (n=42)	0.663*
Posterior tibial artery (n=44)	0.376*
Fibular artery (n=64)	0.265*
Dorsalis pedis artery (n=48)	0.462**

A Spearman’s rho;

* p<0.05;

** p<0.001.

Positive correlations were observed between arteriographic findings and the RI detected for the distal arteries, to *p* < 0.05, for AT, PT, and FIB, and to *p* < 0.001 for DP.

**Figure 1:**
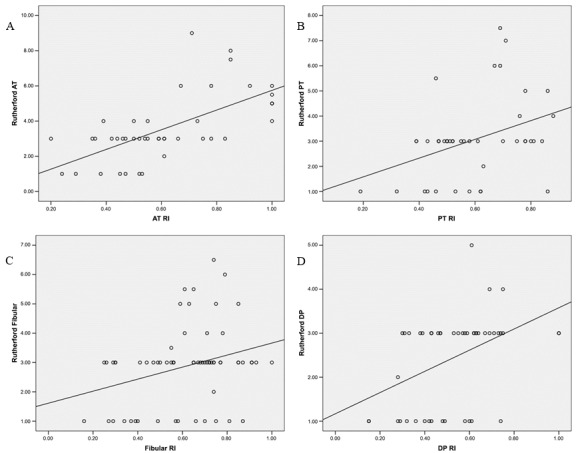
Scatterplots for each distal arterial bed, comparing Rutherford scores with the respective resistance index (RI): **A)** anterior tibial artery (AT); **B)** posterior tibial artery (PT); **C)** fibular artery and **D)** dorsalis pedis artery (DP).

Figure 1 illustrates the distribution of RI values correlated with arteriographic scores for each DAB (AT, PT, FIB, DP), according to the Rutherford classification. In the scatter plots shown in the figure, each point represents an artery and the line corresponds to the correlation trend for the artery in question, i.e., when an artery has a higher Rutherford score it corresponds to a higher RI, both of which indicate a worse DAB.

## DISCUSSION

The present study found a positive correlation between the Rutherford Angiographic Classification and the Resistance Index measured for each distal artery. This finding could validate the RI as a good index for evaluating the DAB, comparing it directly with the DSA. In other words, when the DAB score indicated good runoff (low mean score), the RI found using DU was low (demonstrating lower distal resistance to arterial blood flow). As such, when the Rutherford category classified runoff as good (lower values), vascular resistance was also lower (lower RI).

The Rutherford Angiographic Classification was described in a review article in 1997, in which several classifications were proposed for PAOD and acute LL ischemia that are still used today as references in the global literature.^7^ The Global Vascular Guidelines on the Management of Chronic Limb-Threatening Ischemia, published in 2020, still employ the Rutherford Classification, although the Society of Vascular Surgery also recommends using the Wound, Ischemia, and Foot Infection (WifI) and Global Limb Anatomic Staging System (GLASS) classifications.^5^

The Rutherford Classification does not only assess patency of the arteries of the legs, but also assesses the arterial runoff into the PA at the foot, and is well-established in the literature, in contrast with the GLASS classification, which also assesses the arteries of the foot, but was only presented very recently. For these reasons, the Rutherford Classification was chosen for comparison with the RI of the distal arteries in the present study.^7,19^

Other scores based on arteriography images have been proposed, such as the Trans-Atlantic Inter-Society Consensus Document on Management of Peripheral Arterial Disease II (TASC II),^20^ which only describes the lesions in the arteries and does not classify the distal bed obstructions. In turn, the Bollinger et al. score,^21^ described in 1981, did not initially assess the distal LL arteries and it was only recently, in 2021, that a more complete extension of this classification was presented. ^22^Even then, the anatomic aspects of the plantar arch were not described, so the distal bed cannot be totally assessed with this classification. Neither the TASC nor the Bollinger score adequately assess the arterial bed of the foot.^20-22^

It is possible to visualize the trend for both variables to increase simultaneously in Figure 1, which shows the scatter plots for the AT, PT, FIB, and DP arteries. The continuous lines represent the variables’ rising trend and each artery has a different slope. As such, both the DSA and the RI are capable of detecting the quality of the distal arteries, the first uses an anatomic parameter, with direct visualization, and the second uses a hemodynamic index, without direct visualization of the arteries of the foot.

The study population exhibited considerable homogeneity: a total of 120 patients were studied and exactly half were male. The mean age of the sample was close to 70 years, an age at which the prevalence of CLTI of the LL is higher, as described in the literature. The lifestyle habits and comorbidities of the patients in this study were similar to those described in the literature, with SAH, diabetes mellitus, and chronic smoking the most notable.^23^

Analysis of the distal arteries with the Rutherford classification scored 3.57 (mean) for the AT artery and 2.93 for the FIB artery. Since these arteries can be scored from 1 to 10 (where 10 is the worst DAB), the distal arterial beds assessed were relatively good, which is to be expected, since these were distal arteries that could be revascularized, so they did not have major distal atheromatous involvement. The mean score for the DP artery was 2.44 (possible values are from 0 to 7).

The RI measured for the distal arteries varied from 0.62 for the FIB to 0.52 for the DP, indicating a more dilated and better DAB, but there are no parameters in the literature with values for RI associated with the DAB with which we can compare these results. The RI values can range from 0 to 1.

One limitation of the present study is the fact that although 120 patients were assessed, the number of each type of leg artery was lower. This was because the sample comprised patients with severe arteriopathy who rarely have all three arteries of the leg patent and, most frequently, had only one patent artery or arterial segment.

Another limitation is the lack of postoperative follow-up of the patients to establish the relationship between RI and long-term patency after revascularization surgery. However, the objective of the present study was only to validate RI as a useful index for staging the DAB of distal arteries, by comparing it with the Rutheford Angiographic Classification.^7^

Thus, measurement of the RI of the distal arteries could substitute assessment of the DAB using DSA, since it is an easily applied index that is reproducible and noninvasive and provides objective information on the hemodynamics of the distal LL arteries. Prospective longitudinal studies are needed to assess the patency of revascularizations, relating it to the RI of recipient arteries.

## CONCLUSIONS

In this study, the RIs of the arteries of the leg, measured using Doppler ultrasound, exhibited a positive correlation when compared with the Rutherford Classification. This index could be useful for assessment of the DAB of the LL of patients with chronic limb threatening ischemia.
