# Supplementary material for: Correlation between the vascular resistance index and arteriography for assessment of the distal arterial bed in chronic limb threatening ischemia
Source: J Vasc Bras. 2024 Feb 5;23:e20230071. doi: 10.1590/1677-5449.202300712 (PMC10903956; doi:10.1590/1677-5449.202300712)
Supplement: Translation

# Translation: Correlação entre o índice de resistência vascular e a arteriografia na avaliação do leito arterial distal na isquemia crítica dos membros inferiores

**DOI:** 10.1590/1677-5449.202300711

**Authors:** Gabriela de Oliveira Buril, Esdras Marques Lins, Emmanuelle Tenório Albuquerque Godoi Berenguer de Barros e Silva, Fernanda Appolônio da Rocha, Juliana Cavalcanti de Siqueira Charamba, Rebecca Paes de Andrade Souza Caldas, Isadora Ísis Fernandes Vieira, Paloma Karine Araújo da Silva

**Affiliations:** 1 Universidade Federal de Pernambuco - UFPE, Hospital das Clínicas - HC, Recife, PE, Brasil.; 2 Universidade Federal de Pernambuco - UFPE, Centro de Ciências Médicas - CCM, Recife, PE, Brasil.

**Keywords:** isquemia crônica crítica de membro, resistência vascular, ultrassonografia Doppler, doença arterial obliterante periférica, membros inferiores

## Abstract

**Contexto:**

Pacientes com isquemia crítica (IC) dos membros inferiores (MMII) precisam de arteriografia para o planejamento da cirurgia de revascularização. A ultrassonografia Doppler (UD) não é invasiva e, através da aferição do índice de resistência (IR), pode fornecer informações sobre as artérias distais.

**Objetivos:**

Correlacionar a Classificação Angiográfica de Rutherford com o IR na avaliação do leito arterial distal dos MMII.

**Métodos:**

Estudo transversal, realizado em hospital público terciário, com 120 pacientes portadores de IC dos MMII, entre setembro de 2019 a abril de 2022. Foi comparado o IR das artérias da perna passíveis de serem receptoras de revascularização com a imagem obtida através da arteriografia dessas artérias em acordo com a Classificação Angiográfica de leito distal de Rutherford.

**Resultados:**

Foram avaliados 120 MMII em 120 pacientes com idade média de 68,6 anos. A amostra foi composta de 50,0% de pacientes do sexo masculino. Na amostra, 90,0% pacientes encontravam-se na classe cinco de Rutherford. Os valores do IR encontrados para as artérias de perna apresentaram uma correlação positiva, estatisticamente significativa, quando comparados com a Classificação de Rutherford (tibial anterior, p< 0,01; tibial posterior, p = 0,012 e fibular, p = 0,034 e artéria dorsal do pé, p < 0,001).

**Conclusões:**

Neste estudo, os IRs das artérias da perna obtidos através da ultrassonografia Doppler apresentaram uma correlação positiva quando comparados à classificação de Rutherford. Em pacientes com isquemia crítica, esse índice pode ser útil na avaliação do leito arterial distal dos membros inferiores.

## INTRODUÇÃO

Vários são os exames disponíveis para se avaliar as artérias distais dos membros inferiores (MMII) na doença aterosclerótica obliterante periférica (DAOP). A ultrassonografia com Doppler (UD) costuma ser o exame inicialmente realizado nos pacientes portadores de DAOP^1^ . A seguir, um exame que utilize um meio de contraste pode ser necessário para avaliar mais adequadamente a anatomia das lesões, a saber: angiotomografia, angiorressonância e arteriografia por subtração digital (ASD)^2^ .

Até os dias atuais, a ASD continua sendo o exame padrão-ouro para o planejamento na cirurgia de revascularização dos MMII. Entretanto, a ASD é um exame invasivo, utiliza contraste iodado, expõe o paciente e a equipe médica à radiação ionizante e tem alto custo, além de possuir limitações como a visibilização do leito arterial distal (LAD) a uma oclusão que, por vezes, é inadequada^2-4^ . A visibilização do leito distal pode ser dificultada quando o fluxo sanguíneo fica bastante reduzido, seja por múltiplas e longas oclusões, seja por débito cardíaco insuficiente, seja por importante dor isquêmica que impede que o paciente fique imóvel durante a injeção do meio de contraste. Nessas situações, a visibilização do leito arterial na perna e no pé se torna muito prejudicada^5,6^ .

Com base na ASD, Rutherford propôs uma classificação que tem como objetivo avaliar o LAD para o planejamento da cirurgia de revascularização. Nela, o valor atribuído para cada artéria distal é uma ponderação entre o local da anastomose (local onde a artéria está patente) multiplicado pelo aspecto dessa mesma artéria a jusante, somando-se a isso a ponderação do deságue no pé, através do arco plantar (AP). Um bom leito distal é aquele em que a artéria se encontra patente do local avaliado até um AP completo. Por exemplo, considerando uma artéria tibial anterior (TA) utilizada para anastomose no seu terço distal (valor = 2), patente até o seu deságue no pé, sem estenose maior do que 20% (valor = 0), e um leito arterial do pé em que há continuidade com um vaso patente (valor = 1) e com um AP incompleto (valor = 2), a pontuação calculada para a TA seria a seguinte: **(2 x 0) + (1 x 2) + 1 = 3** ^7^ **.**

Já a UD tem se tornado bastante útil na avaliação da DAOP dos MMII, oferecendo cada vez mais informações. Além de ser um exame de baixo custo, não invasivo, sem exposição à radiação e sem o uso de contraste nefrotóxico, a UD tem a capacidade de avaliar a parede arterial e classificá-la quanto à espessura e à quantidade de cálcio. Além da avaliação local, a UD pode avaliar o leito distal, por meio da aferição do índice de resistência (IR), que estima como se encontra a resistência vascular periférica, ou seja, avalia a resistência ao fluxo na artéria em estudo. Quanto mais baixo for o IR, mais abertas estarão as artérias tronculares distais (melhor o LAD) e mais baixa será a resistência ao fluxo após uma revascularização. A UD tem a vantagem de indicar qual a resistência de uma referida artéria, sem necessariamente ser avaliado morfologicamente todo o leito distal. A principal desvantagem da UD é justamente a dificuldade em avaliar anatomicamente todos os vasos que compõem as artérias do arco plantar, principalmente^1^ . O IR é obtido por uma razão calculada pela diferença entre o pico de velocidade sistólica e a velocidade diastólica final, dividida pelo pico de velocidade sistólica, e avalia indiretamente o que se pode ver na ASD^1,4^ .

É consenso na literatura mundial que a baixa resistência vascular periférica é um dos fatores que mantém a ponte arterial ou a angioplastia abertas. Entretanto, há poucos estudos utilizando a UD para avaliar essa resistência^5^ . Visto que a DAOP dos MMII é um problema de saúde pública crescente, com altos custos para o paciente e para o sistema de saúde, é necessário pesquisar exames menos invasivos e de baixo custo que possam ajudar na decisão terapêutica mais adequada para seus portadores. Sugerindo inclusive, qual artéria deveria ser revascularizada^6-18^ .

O objetivo deste estudo foi correlacionar a Classificação Angiográfica de Rutherford com o IR das artérias distais dos MMII.

## MÉTODO

Foram incluídos no estudo 120 pacientes internados na enfermaria de Cirurgia Vascular do Hospital das Clínicas/Empresa Brasileira de Serviços Hospitalares da Universidade Federal de Pernambuco (HC/EBSERH-UFPE), portadores de IC dos MMII (categoria 4, 5 e 6 de Rutherford^7^ ) e que foram submetidos a UD e ASD das artérias dos membros doentes, para avaliação pré-operatória de uma cirurgia de revascularização, no período de agosto de 2019 a abril de 2022. O modelo de estudo empregado foi o transversal, com coleta de dados prospectiva, e a amostragem utilizada foi a por conveniência, em que todos os pacientes internados com IC dos MMII em pré-operatório da cirurgia de revascularização foram avaliados.

A seleção da amostra foi do tipo censitário, no qual todos os pacientes internados no período do estudo com DAOP e IC dos MMII foram incluídos na pesquisa.

Todos os pacientes foram submetidos a avaliação através da Classificação da DAOP de Rutheford^7^ . Em seguida, os pacientes foram submetidos a UD e, posteriormente, a ASD das artérias dos MMII. A avaliação por UD e das imagens angiográficas foi realizada por um único examinador.

Durante a UD, cada artéria da perna, possível de ser escolhida como sítio receptor da ponte arterial ou da angioplastia, foi avaliada em relação a patência/oclusão, estenoses, calcificações, morfologia de onda de velocidade; além disso, foi realizada aferição da velocidade de pico sistólica (VPS), da velocidade diastólica final (VDF) e do IR. O local dos IR coletados correspondeu ao local onde a artéria distal receptora apresentava um melhor lúmen ou o mais distal possível. Os valores do IR coletados para o LAD a uma oclusão poderiam variar entre 0 e 1,0, sendo 0 correspondente a uma onda de fluxo contínua, e 1,0, a uma onda de alta resistência, sem fluxo diastólico (pior LAD)^17,18^ .

A avaliação das arteriografias foi realizada por observador único. As imagens angiográficas do leito distal foram classificadas de acordo com Rutherford, para que uma análise qualitativa (imagem) pudesse ser transformada numa variável quantitativa. Cada artéria foi avaliada individualmente, mas nem todos os pacientes possuíam todas as três artérias da perna patentes, portanto, o *n* de cada artéria foi diferente e menor do que 120 (Tabelas 1, 2)^7^ .

**Tabela 1:** Ponderação do deságue arterial (total de três unidades) - local^7^ .

**Sítio da anastomose**	**Número de unidades atribuídas**		
**distal (artéria)**	**3**	**2**	**1**
Ilíaca comum		Ilíaca externa	Ilíaca interna
Ilíaca externa	Femoral comum	Femoral	Profunda
Femoral comum		Femoral	Profunda
Poplítea acima do joelho	Poplítea distal		Tibial anterior
Poplítea abaixo do joelho			Tibial posterior
			Fibular
Tibial anterior		Tibial distal	Arco plantar
Tibial posterior		Tibial distal	Arco plantar
Fibular		Deságue podal	Colaterais para as
Dorsal do pé/inframaleolar			artérias tibiais

**Tabela 2:** Ponderação do deságue arterial (total de três unidades) - oclusão^7^ .

**Grau de oclusão**	**Número de pontos atribuídas por unidade**				
	**3**	**2,5**	**2**	**1**	**0**
Vasos de maior deságue	Ocluído em todo o comprimento	Ocluída por menos de ½ da extensão; colaterais visíveis	Maior estenose entre 50% e 99%	Maior estenose entre 20% e 49%	Maior estenose menor do que 20%
Deságue podal	Sem artéria podal patente	Parcialmente pérvio ou totalmente pérvio depois de uma lesão suboclusiva	Continuidade com vaso patente, mas arco incompleto	Um ou mais estenoses suboclusivas	Deságue plantar totalmente patente (estenose <20%)

O valor atribuído a cada artéria foi a ponderação entre o local do potencial anastomose (local onde a artéria estava patente) multiplicado pelo aspecto dessa mesma artéria a jusante. Somava-se a isso a ponderação do deságue podal - arco plantar (AP). Por exemplo, considerando uma artéria tibial anterior (TA) utilizada para anastomose no seu terço distal (valor = 2), patente até o seu deságue no pé́, sem estenose maior do que 20% (valor = 0), e deságue podal em que há continuidade com vaso patente, a artéria dorsal do pé (valor = 1), mas AP incompleto (valor = 2), a pontuação calculada foi a seguinte: TA = (2 x 0) + (1 x 2) + 1 = 3. Todas as equações receberam a soma de uma unidade no fim, para que os resultados fossem sempre maiores do que zero. A pontuação do deságue encontrado para as artérias tibial anterior, tibial posterior e fibular variaram entre 10 e 1, sendo o valor mais alto correspondendo ao pior deságue. Para a artéria dorsal do pé, essa pontuação variou entre 7 e 1^7^ .

A pontuação do deságue encontrado para as artérias TA, tibial posterior (TP) e fibular (FIB), variaram entre 10 e 1, sendo o valor mais alto correspondendo ao pior deságue. Para a artéria dorsal do pé (DP), essa pontuação variou entre 7 e 1^7^ . Nesta pesquisa, não foi realizada uma avaliação isolada da melhor artéria para ser revascularizada. Todas as artérias distais foram estudadas, e o seu respectivo IR foi aferido.

Para análise dos resultados, foi utilizado o software SPSS 13.0 (*Statistical Package for the Social Sciences*) para Windows e o Excel 2010. Todos os testes foram aplicados com intervalo de 95% de confiança. Os resultados foram calculados levando em consideração respostas válidas, ou seja, não foram contabilizadas as respostas ignoradas. Foi utilizado o teste de correlação de Spearman rho para correlacionar as variáveis da Classificação de Rutherford (a pontuação do deságue para as artérias TA, TB e FIB varia entre 10 e 1, sendo o valor mais alto correspondendo ao pior deságue; para a artéria DP, essa pontuação variou entre 7 e 1)^7^ e valores de IR (0,0 a 1,0)^17^ . As variáveis numéricas foram representadas pelas medidas de tendência central e medidas de dispersão. O teste de normalidade de Kolmogorov-Smirnov foi utilizado para variáveis quantitativas.

O estudo foi aprovado pelo Comitê de Ética em Pesquisa do HC/EBSERH-UFPE, sob o registro 3.471.560.

## RESULTADOS

Foram avaliados 120 MMII em 120 pacientes com idade variando entre 32 e 96 anos (média 68,6 anos, com desvio-padrão de 10,3 anos), todos portadores de DAOP por aterosclerose. A amostra foi composta de 60 (50,0%) pacientes do sexo masculino. Na amostra, 108 (90,0%) pacientes encontravam-se na classe cinco de Rutherford (Tabela 3).

**Tabela 3:** Caracterização da amostra de 120 pacientes.

**Características**	**Valor**
**Fatores demográficos**	
Gênero masculino	60 (50,0%)
Idade	68,6 ± 10,3
**Fatores de risco**	
HAS	108 (90,0%)
DM	92 (76,7%)
Tabagismo	61 (50,8%)
IRC HD	8 (6,7%)
**Classificação de Rutherford (DAOP)**	
4	3 (2,5%)
5	109 (90,8%)
6	8 (6,7%)
**Amputação Prévia**	21 (17,5%)
**Tipo de Lesão**	
Espontânea	97 (80,8%)
Traumática	19 (15,8%)

HAS = hipertensão arterial sistêmica; DM = diabetes melito*;* IRC = insuficiência renal crônica; HD = hemodiálise; DAOP = doença aterosclerótica obliterante periférica

Entre as comorbidades analisadas, hipertensão arterial sistêmica (HAS) esteve presente em 108 pacientes (90,0%); DM em 92 pacientes (76,7%); tabagismo em 61 pacientes (50,8%) e 6,7% dos pacientes apresentavam IRC em estágio terminal, com necessidade de hemodiálise.

No quesito amputação, 21 (17,5%) apresentavam algum tipo de amputação prévia; as lesões encontradas nas extremidades foram classificadas como espontâneas em 100 pacientes (83,4%).

A Tabela 3 apresenta a caracterização da amostra estudada. A Tabela 4 mostra a distribuição de pontuação de leito distal, segundo a Classificação de Rutherford^7^ , encontrada em 120 pacientes.

**Tabela 4:** Classificação das artérias do leito distal, segundo Rutherford.

**Artérias (n)**	**Média ± Desvio padrão**	**Mediana (Q1; Q3)**	**Mínimo - Máximo**
TA (42)	3,57 ± 1,92	3,00 (3,00; 4,25)	1,00 - 9,00
TP (44)	3,05 ± 1,61	3,00 (2,25; 3,00)	1,00 - 7,50
FIB (64)	2,93 ± 1,36	3,00 (3,00; 3,00)	1,00 - 6,50
DP (48)	2,44 ± 1,05	3,00 (1,00; 3,00)	1,00 - 5,00

TA = tibial anterior; TP = tibial posterior; FIB = fibular; DP = dorsal do pé

Os IR das artérias distais foi colhido em todas as artérias patentes e passíveis de revascularização. A Tabela 5 mostra a média, a mediana e os valores máximos e mínimos dos IR encontrados para as artérias TA, TP, FIB e DP.

**Tabela 5:** Índices de resistência encontrados nas artérias tibial anterior, tibial posterior, fibular e desvio padrão.

**IR (n)**	**Média ± desvio padrão**	**Mediana (Q1; Q3)**	**Mínimo - máximo**
TA (42)	0,60 ± 0,23	0,57 (0,45; 0,76)	0,20 - 1,00
TP (44)	0,58 ± 0,18	0,56 (0,47; 0,72)	0,19 - 0,88
FIB (64)	0,62 ± 0,20	0,65 (0,49; 0,75)	0,16 - 1,00
DP (48)	0,52 ± 0,19	0,51 (0,39; 0,63)	0,15 - 1,00

IR = índice de resistência; TA = tibial anterior; TP = tibial posterior; FIB = fibular; DP = dorsal do pé.

Depois, a pontuação angiográfica de cada artéria foi correlacionada com seu respectivo IR. A Tabela 6 mostra a correlação entre as Classificação de Rutherford das artérias TA, TP, FIB e DP e os respectivos IR. Foi utilizado o teste de correlação de Spearman rho para análise da correlação entre as variáveis.

**Tabela 6:** Coeficiente de correlação entre a classificação de Rutherford e os índices de resistência das artérias tibiais, fibular e dorsal do pé.

	**Coeficiente de correlação ^A^**
Artéria tibial anterior (n=42)	0,663*
Artéria tibial posterior (n=44)	0,376*
Artéria fibular (n=64)	0,265*
Artéria dorsal do pé (n=48)	0,462**

A Correlação de Spearman rho;

* p<0,05;

** p<0,001

Foi observada correlação positiva entre os achados arteriográficos e o IR detectado nas artérias distais, com *p* < 0,05 para TA, TP e FIB, e *p* < 0,001 para DP.

A Figura 1 demonstra a distribuição dos IR correlacionados com a pontuação arteriográfica de cada LAD (TA, TP, FIB, DP), segundo Rutherford. Nos gráficos de dispersão apresentados na figura, cada ponto corresponde a uma artéria estudada, e a linha corresponde à tendência de correlação de cada artéria, ou seja, quando uma artéria possui uma pontuação de Rutherford maior, corresponde a um IR mais alto - ambos descrevem um pior LAD.

**Figura 1:** Gráficos de dispersão de cada leito arterial distal comparando a pontuação, segundo Rutherford, com o respectivo índice de resistência (IR): **A)** artéria tibial anterior (TA); **B)** artéria tibial posterior (TP); **C)** artéria fibular e **D)** artéria dorsal do pé (DP).

## DISCUSSÃO

Na atual pesquisa, foi encontrada uma correlação positiva entre a Classificação Angiográfica de Rutherford e o IR encontrado para cada artéria distal. Esse achado poderia validar o IR como um bom índice avaliador do LAD, comparando-o diretamente com a ASD. Ou seja, quando a pontuação do LAD demonstrava um bom deságue (baixa média ponderada), o IR encontrado na UD era baixo (demonstrando menor resistência distal ao fluxo sanguíneo arterial). Deste modo, quando o índice de Rutherford classificava o deságue como bom (valores mais baixos), a resistência vascular também foi mais baixa (IR mais baixo).

A Classificação Angiográfica de Rutherford foi descrita em um artigo de revisão de 1997, no qual foram propostas várias classificações para DAOP e isquemia aguda dos MMII, que são utilizadas até hoje como referência pela literatura mundial^7^ . O *Global Vascular Guidelines on the Management of Chronic Limb-Threatening Ischemia*, publicado em 2020, sobre a IC de MMII utiliza ainda a Classificação de Rutherford, apesar de a Society of Vascular Surgery também recomendar o uso das classificações Wound, Ischemia, and Foot Infection (WifI) e Global Limb Anatomic Staging System (GLASS)^5^ .

A Classificação de Rutherford avalia não apenas a patência das artérias das pernas, mas também o deságue arterial no pé através da avaliação do AP, além de estar bem estabelecida na literatura, ao contrário da Classificação GLASS, que também avalia as artérias do pé, mas foi só apresentada muito recentemente. Por esses motivos, a Classificação de Rutherford foi escolhida nesta pesquisa para ser comparada ao IR das artérias distais^7,19^ .

Outros escores baseados em imagens da arteriografia foram propostos, como o Trans-Atlantic Inter-Society Consensus Document on Management of Peripheral Arterial Disease II (TASC II)^20^ , que descreve apenas as lesões das artérias e não classifica o leito distal às obstruções. Já o escore de Bollinger et al.^21^ , descrito em 1981, inicialmente não avaliava as artérias distais dos MMII, sendo que só recentemente, em 2021, uma extensão mais completa dessa classificação foi apresentada^22^ .Ainda assim, nela, os aspectos anatômicos do arco plantar não foram descritos; sendo assim, o leito distal não pode ser totalmente avaliado por essa classificação. Tanto o TASC quanto o escore de Bollinger não avaliam adequadamente o leito arterial no pé^20-22^ .

É possível visibilizar essa tendência em ambas as variáveis que aumentam simultaneamente na Figura 1, onde estão apresentados os gráficos de dispersão para as artérias TA, TP, FIB e DP. As linhas contínuas representam a tendência de aumento das variáveis e têm inclinação diferente para cada artéria. Sendo assim, tanto a ASD quanto o IR têm a capacidade de detectar a qualidade das artérias distais, sendo que aquela utiliza um parâmetro anatômico, com visibilização direta, e este utiliza um índice hemodinâmico, sem visibilização direta das artérias do pé.

A população estudada se mostrou bastante homogênea: foram estudados 120 pacientes, e exatamente a metade era composta de indivíduos do gênero masculino. A amostra possuiu idade média de quase 70 anos, idade em que a prevalência de IC dos MMII é maior, como descrito na literatura. Quanto aos hábitos de vida e as comorbidades encontradas nos pacientes desta pesquisa, foram semelhantes às descritas na literatura, merecendo destaque a HAS, o diabetes melito e o tabagismo crônico^23^ .

A análise das artérias distais segundo Rutherford mostrou que as pontuações recebidas pelas artérias foram 3,57 (média) para artéria TA e 2,93 para a artéria FIB. Como essas artérias poderiam ter pontuações que variam entre 1 e 10 (sendo 10 o pior LAD), os leitos arteriais distais encontrados foram relativamente bons, o que era de se esperar, pois foram avaliadas artérias distais passíveis de serem revascularizadas, portanto, que não tiveram grande acometimento ateromatoso distal. Para a artéria DP, a média das pontuações encontrada foi de 2,44 (os valores poderiam variar de 0 a 7).

Quando se avaliaram os IR colhidos para as artérias distais, os valores variaram entre 0,62 para a FIB e 0,52 para a DP, indicando LAD mais dilatado e melhor LAD, mas não há na literatura um parâmetro, com valores de IR associado com o LAD, para que possamos comparar. Os valores de IR poderiam variar entre 0 e 1

Uma limitação do atual estudo foi o fato de que, apesar de terem sido avaliados 120 pacientes, quando foram consideradas as artérias da perna separadamente, o número isolado de cada uma delas foi menor. Isso aconteceu porque foram estudados pacientes arteriopatas graves, que raramente possuíam as três artérias da perna patentes e, mais frequentemente, apresentavam apenas uma artéria, ou segmento de artéria, patente.

Outra limitação a falta de um acompanhamento dos pacientes operados para estabelecer uma relação entre o valor do IR e a patência em longo prazo da cirurgia de revascularização. Porém, o objetivo da atual pesquisa foi apenas validar o IR como um índice útil para estudar o LAD das artérias distais, através da sua comparação com a Classificação Angiográfica de Rutheford^7^ .

Assim, a aferição do IR das artérias distais poderia substituir a avaliação do LAD através da ASD, já que se trata de um índice de fácil realização e reprodutibilidade, não invasivo, que dá informações objetivas da hemodinâmica das artérias distais dos MMII. Estudos prospectivos longitudinais são necessários para avaliar a patência das revascularizações, relacionadas ao IR das artérias receptoras.

## CONCLUSÃO

Neste estudo, os IRs das artérias da perna, obtidos através da ultrassonografia Doppler, apresentaram uma correlação positiva quando comparados à classificação de Rutherford. Em pacientes com isquemia crítica, esse índice pode ser útil na avaliação do LAD dos MMII.
